# Comparison of clinical and radiological outcomes between modified Gallie graft fusion-wiring technique and posterior cervical screw constructs for Type II odontoid fractures

**DOI:** 10.1097/MD.0000000000011452

**Published:** 2018-07-20

**Authors:** Hui Wang, Rui Xue, Lumei Wu, Wenyuan Ding, Lei Ma

**Affiliations:** Department of Spine Surgery, The Third Hospital of HeBei Medical University, Shijiazhuang, China.

**Keywords:** modified Gallie graft fusion-wiring technique, posterior cervical screw constructs, Type II odontoid fractures

## Abstract

The aim of this study was to compare clinical and radiological outcomes between modified Gallie graft fusion-wiring technique and posterior cervical screw constructs for Type II odontoid fractures, and hope to provide references in decision making and surgical planning for both spinal surgeons and surgically treated patients.

This is a retrospective study. By retrieving the medical records from January 2005 to July 2015 in our hospital, 53 Type II odontoid fracture patients were reviewed. According to the instrumentation type, patients were divided into 2 groups: Wiring group and Screw group. Three categorized factors were analyzed statistically: patient characteristics: age, body mass index, preoperative neurological status, duration, complicated injuries; surgical variables: surgery time, blood loss, vertebral artery injury, spinal cord or nerve root injury, major systemic complications, wound infection, pain at the bone donor area, instrumentation failure, revision rate; and radiographic parameters: preoperative and final follow-up data of C_0–2_ curvature, C_2–7_ curvature, C2-C7 sagittal vertical axis, C7 slope, fracture classification, separation, and displacement of odontoid fracture, fusion rate. An additional comparison of surgical outcomes was done, including patient satisfaction, visual analog scale score for neck pain, neck stiffness, medical expense.

There was no statistically significant difference between the 2 groups in patient characteristics of age, sex, body mass index, preoperative neurological status, duration, and complicated injuries. No statistically significant difference was noted in surgical variables of blood loss, vertebral artery injury, spinal cord or nerve root injury, major systemic complications, wound infection, bone harvested zone pain, instrumentation failure, revision rate. The surgery time was shorter in Wiring group than that in Screw group, with a statistically significant difference. We noted no significant difference between the 2 groups when comparing radiographic parameters of preoperative and final follow-up data of C_0–2_ curvature, C2-C7 sagittal vertical axis, fracture classification, the separation and displacement of odontoid fracture, and fusion rate. Although we noted no significant difference in preoperative C_2–7_ curvature and C7 slope, the final follow-up data showed that C_2–7_ curvature and C7 slope were smaller in Wiring group than that in Screw group. We noted no significant difference in visual analog scale score, neck stiffness, and neurological status at final follow-up. The medical expense was less in Wiring group; the patient satisfaction was lower in the Wiring group than that in the Screw group.

The modified Gallie graft fusion-wiring technique provided solid fusion and stabilization for patients with Type II odontoid fractures, Gallie graft fusion-wiring resulted in less surgery time, less medical expense, but lower patient satisfaction when compared with the posterior cervical screw constructs.

## 1 Introduction

Anderson and D’Alonzo type II fractures of the odontoid process are not rare, and the mechanism is generally suggested to be hyperflexion or hyperextension of the cervical spine.^[1–3]^ Although most Type II odontoid fractures can be managed either with conservative treatment (immobilization) or with surgical intervention (anterior odontoid screw fixation). There are several subsets that are not amendable to these treatment measures, such as odontoid fractures with a significant displacement, with oblique fracture line in the frontal plane that precludes anterior odontoid screw placement, with a large thoracic kyphosis or a very large barrel chest that precludes the appropriate angle for anterior odontoid screw placement, and with a ruptured transverse ligament.^[4,5]^

Posterior fixation of C1 lateral mass screws combined with C2 pedicle screws has become the final alternative of posterior C1-C2 fixation with a low incidence of complication.^[5–7]^ The procedure is technically demanding steep learning curve and an exact 3-dimensional understanding of the anatomy of the region, in case the high potential risk of vertebral artery (VA) and spinal cord injury.^[8,9]^ Moreover, VA injuries could happen during subperiosteal exposure of C1 posterior ring due to atypical VA loop. Regarding C1 lateral mass screw placement, there is a risk of damaging the internal carotid artery and the hypoglossal nerve, if anterior surface of C1 lateral mass is penetrated.^[10,11]^

Posterior cervical wiring of the lamina of C1 and C2 dates to 1939 by Gallie.^[12]^ Dickman et al^[13]^ modified the posterior wiring technique by adding bone fusion between C1 and C2 arches in 1991, and recommends the use of a halo to immobilize patients for 3 months after surgery and the use of a rigid cervical collar for an additional 1 to 2 months after that. With this kind of immobilization, they have reported a 97% fusion rate with the technique.^[13]^ However, graft breakage and wire loosening are relatively common complications of wiring technique.^[14]^ With the modification and optimization of the fixed material, the strength and flexibility of the current stainless cable have improved, and is superior in fatigue life when compared with traditional ones, but received little attention due to the limited use of the posterior wiring technique.

In the current study, we compare the clinical and radiological outcomes between modified Gallie graft fusion-wiring technique and posterior cervical screw constructs for Type II odontoid fractures, and hope to provide references in decision making and surgical planning for both spinal surgeons and surgical treated patients.

## 2 Materials and methods

### 2.1 Patients

This is a retrospective study and was approved by the Institutional Review Board of the Third Hospital of Hebei Medical University before data collection and analysis (2017–0035), each patient provided informed consent. The inclusion criteria included Type II C odontoid fractures that were operated on by posterior C1/C2 fusion and stabilization; Grauer type IIA and IIB fractures combined with anterior translation that could not be reduced or fixed via an anterior approach; Patients were available for a final assessment at a minimum of 1-year postsurgery; and complete radiographic data, include cervical anteroposterior, lateral, transoral X-ray at preoperative, immediate postoperative, 12 months, and annually thereafter postoperatively, and computed tomography (CT) at final follow-up. Exclusion criteria included Anderson and D’Alonzo type I fractures, type III fractures; combined with Jefferson fracture, Hangman fracture, lower cervical fracture; and rheumatoid atlantoaxial instability.

By retrieving the medical records from January 2005 to July 2015 in our hospital, 53 patients who met both the inclusion and exclusion criteria were retrospectively reviewed: 18 F and 35M, with a mean age of 35.8 ± 11.9 years (range from 23 to 66 years). Overall, 37 patients experienced traffic accident, 11 patients fell from a height, and 5 patients got hurt in fights. Duration from injury to operation range from 1 day to 3 weeks. According to the classification, 12 patients of Grauer type IIA, 10 patients of Grauer type IIB, 31 patients of Grauer type IIC, 7 patients complicated by head injury, 7 patients by thoracic trauma, 4 patients by limb fracture. Reduction of the displaced odontoid fracture was attempted using the Mayfield head-holding device and appropriate cervical positioning on the Jackson table preoperatively. In most cases, fracture alignment improved, but complete anatomic reduction of the odontoid was not achieved. According to the instrumentation for fixation, patients were divided into 2 groups: Wiring group (17 patients) and Screw group (36 patients).

### 2.2 Surgical strategy

2.2.1 Modified Gallie wiring technique

**Figure 1:**
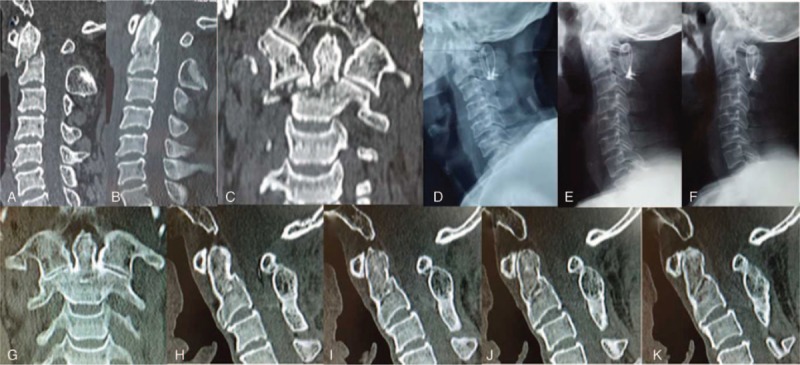
Male, 35 years old, duration from injury to operation was 5 days. (A) Preoperative CT showed type II C odontoid fracture, with fracture anterior displacement = 7.3 mm. (B, C) Reduction of the displaced odontoid fracture was attempted using the Mayfield head-holding device. (D–F) Postoperative lateral, flexion, and extension X-ray showed the cervical alignment and mobility was excellent after modified Gallie graft fusion-wiring instrumentation. (G–K) Twelve-month follow-up CT scan showed evident bridging bone across the odontoid fracture site in coronal and sagittal CT-scans in neutral head position.

All patients received awake intubation, and the surgical position was prone, taking care to avoid excessive pressure on the eyes, with intraoperative gravity traction. The incisions were at midline; infiltration of the skin and subcutaneous tissue with a dilute 1:500,000 epinephrine solution is helpful to provide hemostasis. Using electrocautery and elevators, we exposed the posterior elements subperiosteally and inserted self-retaining retractors. A sublaminar cable was passed under the posterior C1 arch from inferior to superior. Both the superior aspect of the C2 spinous process and the inferior arch of C1 are decorticated before graft placement. Next, a notched iliac crest approximately 3 cm long with a caudal notch for the C2 spinous process is placed in between the spinous process of C2 and wedged underneath the posterior arch of C1. All patients wore a cervical collar postoperatively for at least 3 months, and took off after confirmation of bone fusion on CT (case see Fig. 1).

2.2 Posterior cervical screw technique

**Figure 2:**
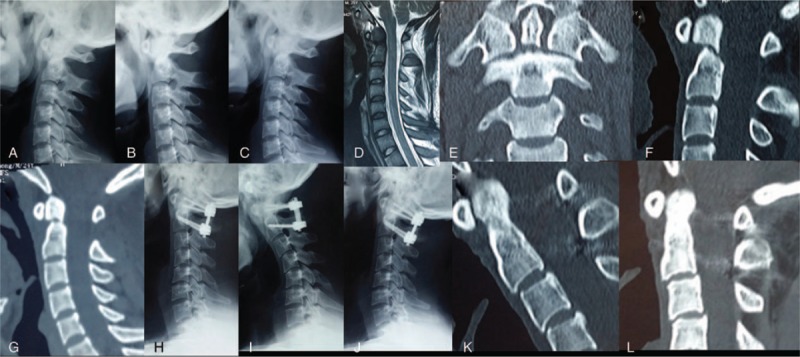
Male, 29 years old, duration from injury to operation was 8 days. (A–C) Preoperative lateral, flexion, and extension X-ray showed odontoid fracture. (D) Preoperative MRI showed odontoid fracture without spinal cord compression. (E, F) Preoperative CT showed type II B odontoid fracture, with fracture anterior displacement = 8.1 mm. (G) Reduction of the displaced odontoid fracture was attempted using the Mayfield head-holding device. (H–J) Postoperative lateral, flexion, and extension X-ray showed posterior C1 lateral screw-C2 pedicle screws fixation. (K) Three-month follow-up CT scan showed bridging bone across the odontoid fracture site in sagittal CT-scans in neutral head position. (I) Twelve-month follow-up CT scan showed evident bridging bone across the odontoid fracture site in sagittal CT-scans in neutral head position.

All patients received awake intubation, and the surgical position was prone, taking care to avoid excessive pressure on the eyes, with intraoperative gravity traction. The incisions were at midline; infiltration of the skin and subcutaneous tissue with a dilute 1:500,000 epinephrine solution is helpful to provide hemostasis. Using electrocautery and elevators, we exposed the posterior elements subperiosteally and inserted self-retaining retractors. The C1 lateral mass screws were inserted at the crossing of the inferior rim of the posterior arch and the middle of the lateral mass, aiming at the center with a cephalad angulation of 20°. We placed C2 pedicle screws in the upper outer quadrant of the C2 lateral mass and were angulated approximately 20° medially and superiorly. The C1 lateral mass screws and C2 pedicle screws were linked with rods bilaterally; an autologous iliac bone graft was inserted between the posterior arch of C1 and the laminae and spinous process of C2 for fusion. All patients wore a cervical collar postoperatively for 6 to 8 weeks (case see Fig. 2).

### 2.3 Clinical and radiological evaluation

**Figure 3:**
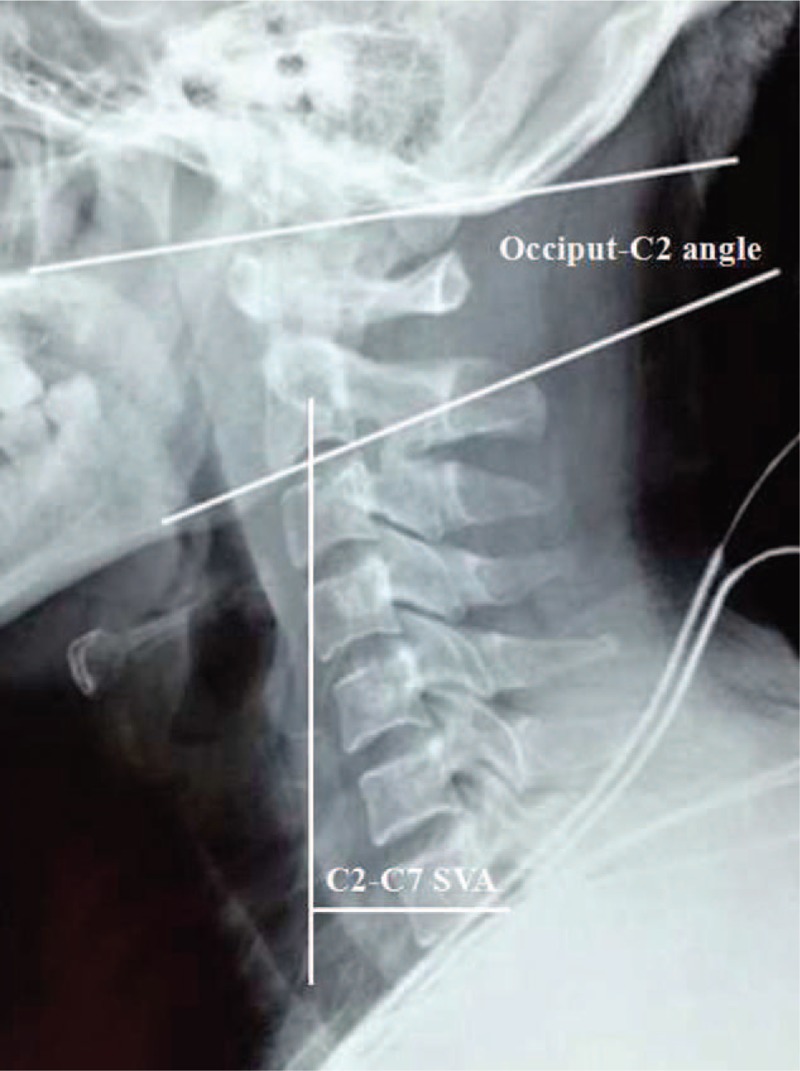
C0–2 Cobb angle was measured as the angle between the McGregor line and lower endplate of the C2 vertebra. A positive value indicates lordosis between the occiput and C2, and a negative value indicates kyphosis between the occiput and C2. C2-C7 sagittal vertical axis (SVA) was the horizontal distance between the C2 plumb line and the posterior corner of C7.

**Figure 4:**
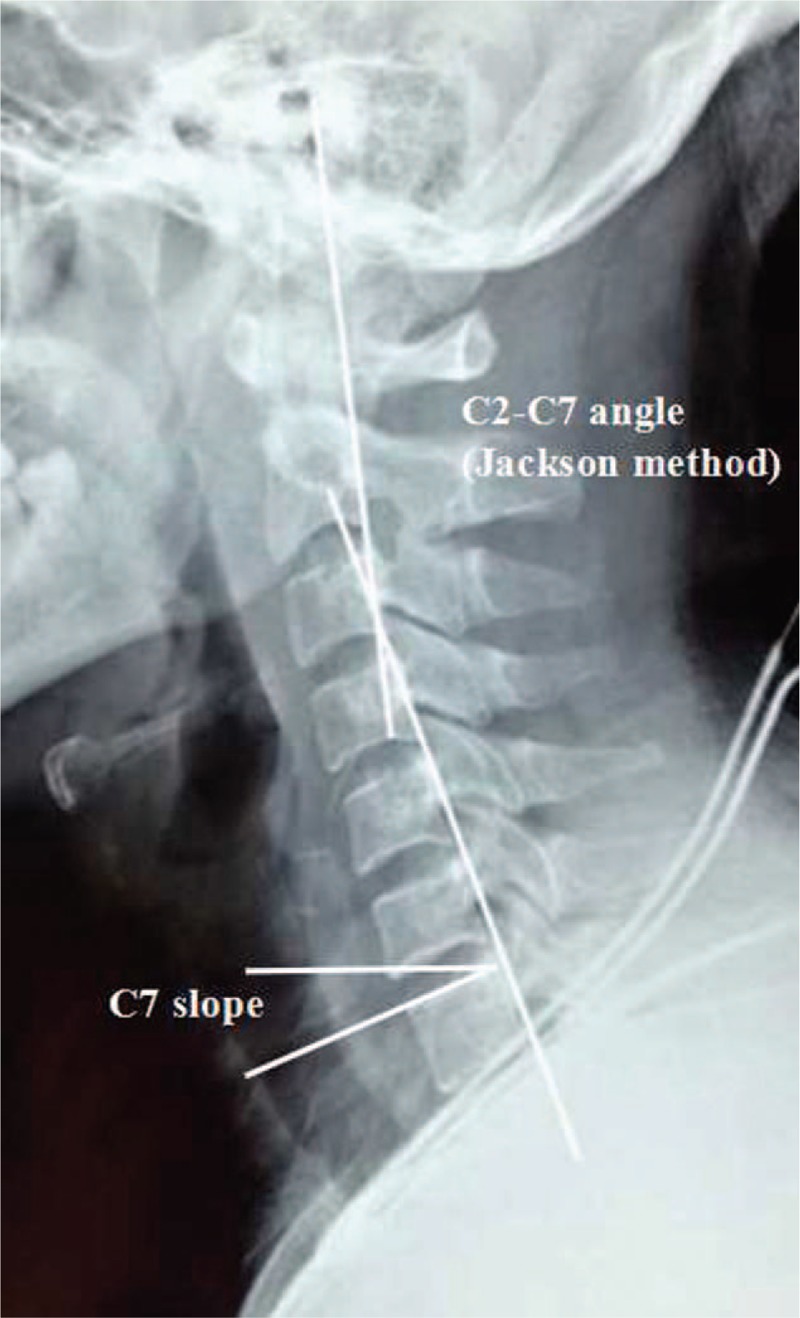
C2–7 Cobb angle (Jackson method) was measured at the intersection of the posterior body tangent lines on C2 and C7. C7 slope was defined as an angle formed between the C7 upper end plate and the horizontal plane.

**Figure 5:**
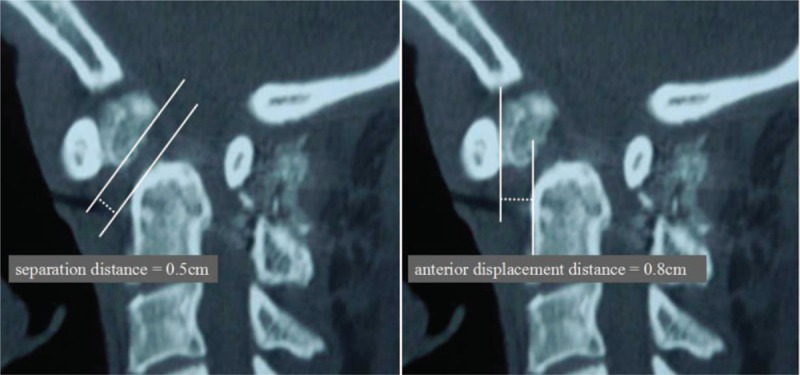
Separation and displacement of odontoid fracture.

**Figure 6:**
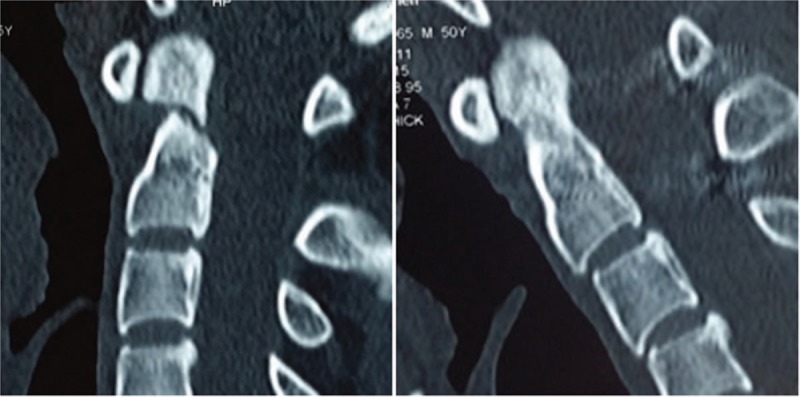
Fracture healing was defined as evident bridging bone across the odontoid fracture site on 1 of the cortices in sagittal CT-scans in neutral head position.

To investigate the difference of clinical and radiological outcomes between Wiring group and Screw group, 3 categorized factors were analyzed statistically: patient characteristics: age at operation, sex, body mass index (BMI), preoperative neurological status evaluated by Japanese Orthopaedic Association Scores (JOA), duration (from injury to operation), complicated injuries (head injury, thoracic trauma, limb fracture); surgical variables: surgery time, blood loss, VA injury, spinal cord or nerve root injury, major systemic complications (respiratory, cardiovascular, urinary, deep vein thrombosis, pulmonary embolism), wound infection, pain at the bone donor area, instrumentation failure, revision rate; and radiographic parameters: preoperative and final follow-up data of C0–2 curvature, C2-C7 sagittal vertical axis (SVA) (Fig. 3), C2–7 curvature, C7 slope (Fig. 4), fracture classification, separation and displacement of odontoid fracture (Fig. 5), and fusion rate defined as evident bridging bone across the odontoid fracture site on one of the cortices in sagittal CT scans in neutral head position (Fig. 6). Additional comparison of surgical outcomes, including patient satisfaction, visual analog scale score for neck pain (VASSNP),^[15]^ and neck stiffness were performed between the 2 groups. The patients’ satisfaction was evaluated by the modified Patient Satisfaction Index (m-PSI) at final follow-up, with response of 1 or 2 indicating a satisfactory outcome and a PSI response of 3 or 4 indicating an unsatisfactory outcome (Table 1). Patients were followed up in an outpatient clinic after initial treatment. JOA, VASSNP, neck stiffness (none/mild/ severe),^[16]^ and patient satisfaction at the time of final follow-up were recorded.^[16]^ Medical expense was also recorded and compared between the 2 groups.

**Table 1:**
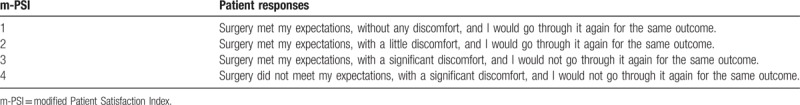
Modified Patient Satisfaction Index (m-PSI)^[16]^.

### 2.4 Statistical analysis

Data were analyzed using Statistical Product and Service Solutions software (version 13; SPSS, Chicago, IL). Continuous variables were measured as mean ± standard deviation, and categorical variables were expressed as frequency or percentages. An independent *t* test was used to analyze the difference of continuous variables between two groups. A χ^2^ analysis and Fisher exact test were used to examine the differences among categorical variables. Variables with *P* values smaller than .05 were considered to be of significant difference.

## 3 Results

There was no statistically significant difference between the Wiring group and Screw group in patient characteristics such as age, sex, BMI, preoperative neurological status, duration from onset of the injury to operation, and complicated injuries (Table 2).

**Table 2:**
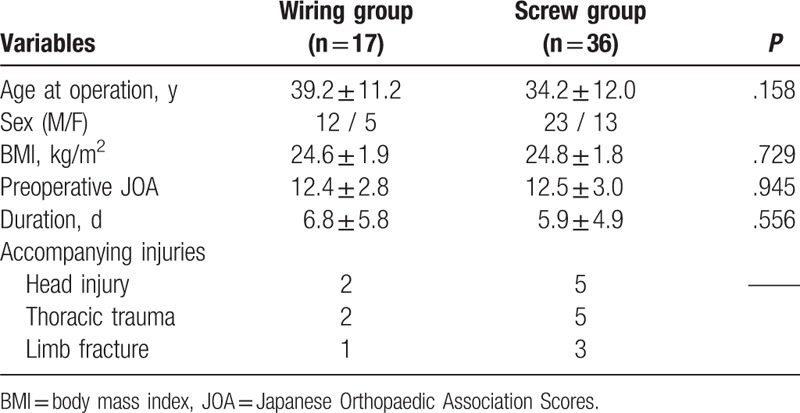
Comparison of patient characteristics between Wiring and Screw groups.

**Figure 7:**
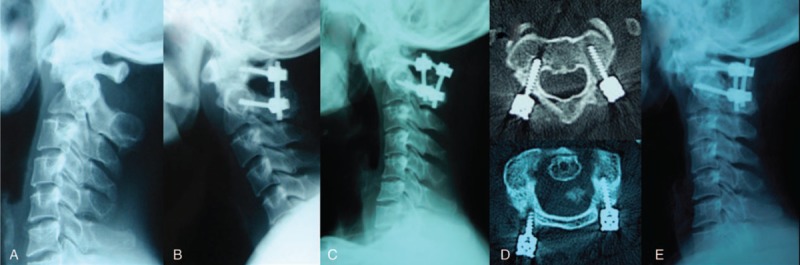
(A) Preoperative lateral X-ray showed C2 fracture, with Geier-deformity. (B) Postoperative lateral X-ray showed C1 lateral mass screws with C2 pedicle screws. (C) C1 lateral mass screw loosening 11 months postoperatively. (D) CT showed C1 lateral mass screw loosening bilaterally. (E) Revision surgery with new C1 lateral mass screw reimplantation.

There was no statistically significant difference between the Wiring group and Screw group in surgical variables of blood loss, VA injury, spinal cord or nerve root injury, major systemic complications, wound infection, bone harvested zone pain, instrumentation failure, and revision rate. The surgery time was shorter in the Wiring group when compared with the Screw group, with a statistically significant difference (Table 3). In the Screw group, 1 patient experienced C1 lateral mass screw loosening 11 months postoperatively and received revision surgery (Fig. 7).

**Table 3:**
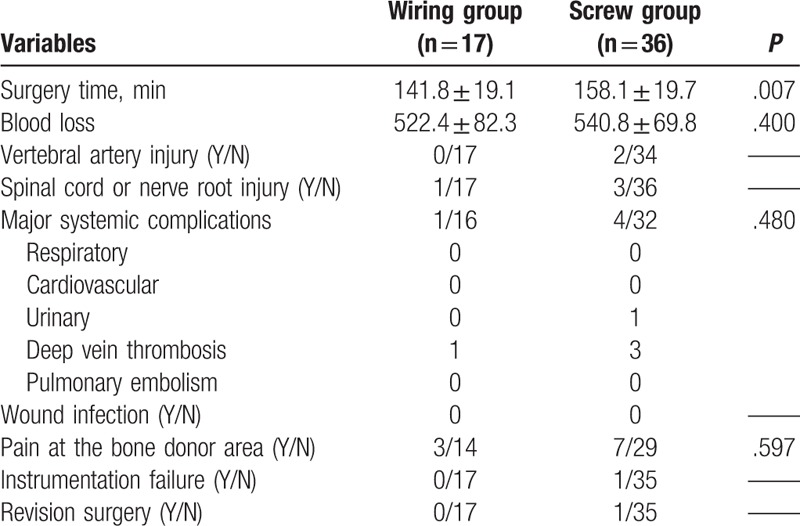
Comparison of surgical variables between Wiring and Screw groups.

There was no statistically significant difference between the Wiring group and Screw group in radiographic parameters of preoperative and final follow-up data of C_0–2_ curvature, C2-C7 SVA, fracture classification, the separation and displacement of odontoid fracture, and fusion rate. Although no significant difference was observed in preoperative C_2–7_ curvature and C7 slope between the 2 groups, the final follow-up data showed that C_2–7_ curvature and C7 slope were smaller in Wiring group than that in Screw group, with a statistically significant difference (Table 4).

**Table 4:**
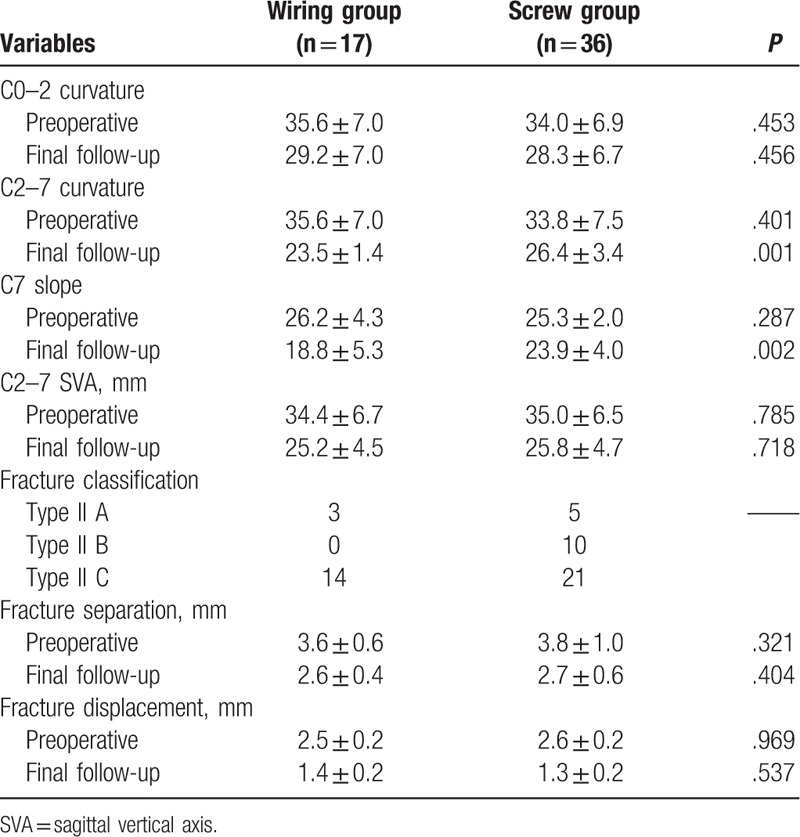
Comparison of radiographic parameters between Wiring and Screw groups.

There was no statistically significant difference between the Wiring group and Screw group in visual analog scale score for neck pain, neck stiffness, and neurological status at final follow-up. The medical expense was less in the Wiring group than that in the Screw group, with a statistically significant difference. Moreover, the patient satisfaction was lower in the Wiring group than that in the Screw group, with a statistically significant difference (Table 5).

**Table 5:**
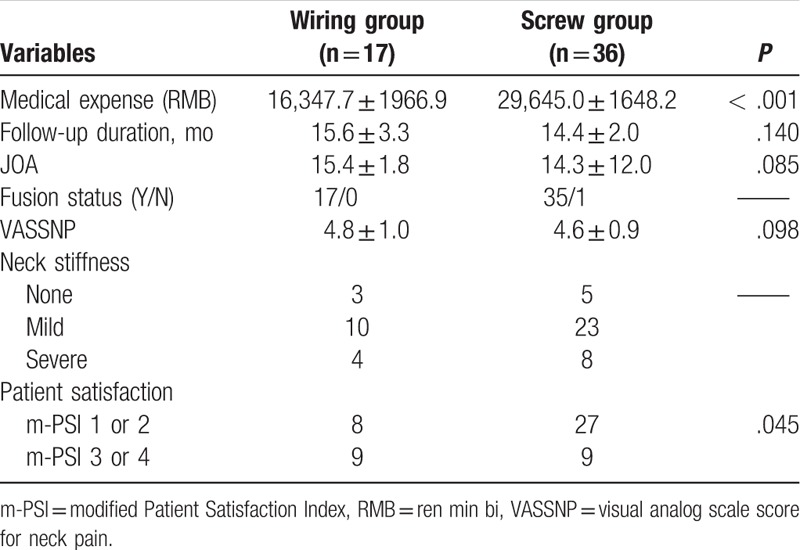
Comparison of surgical outcome at final follow-up between Wiring and Screw groups.

## 4 Discussion

In the current study, the surgery time was shorter in Wiring group when compared with Screw group; this difference may be due to the surgical technique characteristics individually. Goel and Laheri^[17]^ performed the first C1–2 fusion in 1988; both the C1 lateral mass and C2 pedicle screw were placed monocortically over a steel plate.^[18]^ The screw implantation is a high-risk procedure, as VA injury or spinal cord injury may occur due to mal-positioned screw. Although the screw implantation technique has been clearly described in the literature. The C1 lateral mass screws are inserted at the crossing of the inferior rim of the posterior arch and the middle of the lateral mass, aiming at the center with a cephalad angulation of 20°. C2 pedicle screws enter in the upper outer quadrant of the C2 pars and are angulated approximately 20° medially and superiorly.^[17,19,20]^ However, identification of the anatomical structures does not always work, because actual intraoperative entry point and trajectory of the screws may alter due to the varied surgical position, surgeons’ experience, fracture displacement, and intraoperative weight traction. Accurate screws implantation requires repetitive intraoperative C-arm X-ray guidance and confirmation as well, which may increase surgery time and radiation exposure. The traditional “Gallie method” of C1–2 wiring and grafting after scraping the atlantoaxial joints resulted in a failure rate of 80%.^[21]^ Dickman et al^[13]^ modified the Gallie graft fusion technique in the early 1990s in an attempt to improve stability of the construct and avoid bilateral C2 sublaminar cables. They decorticated the contact surfaces of C1 and C2 arches and interposed curvilinear strut graft approximately 4 cm long with a caudal notch for the C2 spinous process, which provided extra stability. The graft was then fixed by a cable passing under the posterior arch of C1 and looped around a notched inferior C2 spinous process, enhancing the stability of the graft. The modified technique is a relatively simple procedure, with a negligible risk of injury to the VA and a very limited impact of anatomical variations of the C2 pedicle.^[22]^

Posterior wiring techniques have long been the priority of surgical stabilization of atlantoaxial complex. There are advantages as well as disadvantages of modified Gallie graft fusion-wiring technique (modification by Dickman et al^[13]^). The advantages include simple to apply, valuable addict to other firmer fusion methods, and can be a salvage procedure (pedicle is small or AV injury during screw technique). Disadvantages of posterior wiring techniques are related to the fact that these techniques can only be applied safely when posterior elements are intact, and the bone quality of arches is adequate.^[22,23]^ Another drawback of posterior wiring technique is the prolonged period of hard external support and that the cable has to be passed below the arch of C1, which carries a risk for spinal cord injury.^[24]^ They are relatively easy but of limited stiffness. In particular, they cannot reach the equivalent stability in translation and rotation when compared with other screw construct techniques. We found that reduction of fracture separation and displacement was satisfactory; bone fusion was achieved in all the patients. We noted no significant differences in neck pain, neck stiffness, and neurological outcome between the 2 treatment groups at final follow-up. We hypothesize that 2 facts are responsible for the good outcome of modified Gallie graft fusion-wiring technique. First, contact surfaces decortication of C1 and C2 arches and interposed curvilinear strut graft with a caudal notch for the C2 spinous process are critical technical points for postoperative high bone fusion rate. Second, all patients were asked to wear a cervical collar postoperatively for at least 3 months and were allowed to take off after confirmation of bone fusion on CT, which could provide long-term stabilization of atlantoaxial complex. However, according to the m-PSI, the patient satisfaction was lower in Wiring group than that in Screw group, though the medical expense was less in Wiring group. It seems that the patient satisfaction does not directly relate to the medical expense. Among the unsatisfied patients, long-term cervical collar immobilization is the main complaint in the current study.

Maximilian et al^[19]^ observed a characteristic cervical spine deformity in geriatric patients with type II odontoid fractures and termed it the “Geier-deformity”; clinical findings include sagittal imbalance and kyphosis of the lower cervical spine, resulting in a loss of the physiological alignment and stooped forward posture. In the current study, C_0–2_ curvature, C_2–7_ SVA, C_2–7_ curvature, and C7 slope decreased from preoperative to final follow-up in both the 2 groups, which depicted the decrease of the cervical lordosis in lower cervical alignment. C_2–7_ curvature and C7 slope were smaller in Wiring group than that in Screw group at the final follow-up; this difference may be due to the surgical technique characteristics individually. For the modified Gallie wiring technique involving a cable passed the posterior arch of C1 and looped around a notched inferior C2 spinous process, tightening the cable is required to make the interface between graft-bone and grafting bed closer, thereafter increasing the regional lordosis in atlantoaxial complex and decreasing the lower cervical lordosis as compensation.^[25,26]^ For the posterior cervical screw technique, rod-screw fixation in situ is enough to provide stabilization, without the need to provide extra compression.

There are some limitations to this study. First, this was a single-center study and only 53 patients were enrolled, and selection bias may exist. Second, the study was conducted retrospectively by case selection, and was not randomized and controlled. Even with these issues in this study, we hypothesize that the modified Gallie graft fusion-wiring technique could provide solid fusion and stabilization for patients with Type II odontoid fractures, when compared with the posterior cervical screw constructs.

## Author contributions

**Conceptualization:** lei ma.

**Data curation:** hui wang, rui xue, lumei wu.

**Formal analysis:** rui xue.

**Software:** lumei wu.

**Writing – original draft:** hui wang.

**Writing – review & editing:** wenyuan ding, lei ma.
